# Hierarchical enhanced non‐rigid registration for target volume correction and propagation for adaptive external beam radiotherapy of carcinoma of the prostate

**DOI:** 10.1120/jacmp.v14i5.4374

**Published:** 2013-09-06

**Authors:** Adrian Andronache, Jerome Krayenbuehl, Gabor Szekely, Ilja F. Ciernik

**Affiliations:** ^1^ Computer Vision Laboratory Federal Institute of Technology Zurich Switzerland; ^2^ Department of Radiation Oncology Zurich University Hospital Zurich Switzerland; ^3^ Department of Radiation Oncology City Hospital Dessau Germany; ^4^ Center for Clinical Research Zurich University Hospital Switzerland

**Keywords:** adaptive radiotherapy, external radiotherapy, prostate cancer, non‐rigid registration

## Abstract

Volumes change during fractionated radiotherapy (RT). We investigate a tool based on the Hierarchical Enhanced Registration Algorithm (HERA) to project a 3D segmentation set of the prostate into the subsequent imaging sets at any time point during RT by using intensity‐based image registration techniques. Sequential CT sets during RT at 15, 30, 45, and 60 Gy of two patients were used. Five expert clinicians outlined the prostate in a blinded fashion, defining intraobserver and interobserver variability on a set of 35 and 25 scans, respectively. The observer variability and positioning for manual correction was compared to both affine and elastic image registration‐based contour propagation. The overall mean error of the registration‐based correction of the planning target volume was comparable to the interobserver variability of manual target volume definition. The correction by affine image fusion was inferior to the results of elastic registration. The maximal deviation for the interobserver segmentation was 15.4 mm, 10.5 mm for the affine and 8.0 mm for the elastic registration. The mean interobserver variability was 1.5 (± 1.4) mm, 2.8 (± 2.3) mm for the affine, and 2.2 (± 1.9) mm for the elastic registration. Intensity‐based elastic registration of deformable anatomical structures with HERA is suitable for the assessment of changes of prostate volumes for the purpose of target propagation and adaptive radiotherapy.

PACS number: 87.57.nj

## I. INTRODUCTION

External beam radiotherapy of the prostate is generally performed over a larger number of fractions. Over this period, the volumes of the rectum and bladder can fluctuate causing displacement and may contribute to the deformation of the target volumes, as well. Therefore, anatomical and geometrical uncertainties must be taken into account by the planning target volume (PTV). The spatial plasticity of the prostate during the course of RT has been well documented, and the volume of the prostate may slightly increase during the first days of RT and shrink during the following weeks.^(^ ^1^ ^,^ ^2^ ^)^ In order to keep PTV margins minimal, daily target positioning verification before treatment can be beneficial.^(3)^ Several techniques have been investigated to account for the intertreatment positioning variability of the prostate. Before high‐resolution in‐room fluoroscopy or 3D on‐board imaging systems became available, the localization of the prostate mostly relied on remote anatomical bony markers. Endorectal spacers help to predict the position of the prostate and could reduce internal target volume (ITV) margins.^(4)^ Direct prostate localization is achieved with fiducial markers in the prostate.^(5)^ Other positioning systems rely on ultrasound technology or transponders.^(^ ^6^ ^,^ ^7^ ^)^ All these approaches, however, neglect the plasticity of the target structures and volumes over the treatment time.

On‐board CTs during RT provide a novel opportunity to redefine the volume of the prostate on a daily basis and, if necessary, adapt the CTV. However, the reliability of CT‐based prostate localization and volume assessment may be reduced by technical limitations, such as the typical low contrast soft tissue differentiation of the common on‐board CT imaging systems, interfering with resegmentation and automated target volume reconstruction.

In the present work, we investigate the possibility of using image registration techniques as a tool for target volume propagation. Image registration is the process of transforming the different sets of data onto one coordinate system, such that the scene or the objects pictured from different views and/or at different time points are brought into alignment. Considering that the physician can make a detailed planning and contouring of the prostate onto an initial CT scan of a patient, we registered it to all the subsequent CTs acquired during the RT. By using nonrigid image registration techniques such as the Hierarchical Enhanced Registration Algorithm (HERA),^(8)^ we studied the performance of both affine and nonrigid transformations to estimate the spatial changes and to compensate for the plasticity of the pelvic structures. The information from the positioning changes of the pelvis is applied to the prostate clinical target volume (CTV), allowing precise prostate localization during potentially each single treatment sessions. The ability of the physicians to manually define the prostate volume for the purpose of adaptive radiotherapy is compared to the performance of the proposed automated computer‐based CTV registration. Routine adaptation of the prostate volume and online replanning to account for volume changes during radiotherapy has not been introduced in routine treatment yet. One major requirement in implementation of adaptive image‐guided radiotherapy will rely on optimal iterative target volume assessment procedure. Using a method based on ray casting, hierarchical image registration and manual segmentation is a novel approach in the context of adaptive radiotherapy. We hypothesize that the automated registration with HERA will improve the contouring of the prostate and eventually allow minimizing PTV margins for the purpose of adaptive radiotherapy, especially posteriorly, avoiding underdosing the posterior lobes of the prostate.

## II. MATERIALS AND METHODS

### A. Patient characteristics and CT acquisition

Sequential CTs were obtained from two prostate cancer patients undergoing external beam radiotherapy. Patients were scanned and treated in a supine position with a rectal balloon with $40\;{\text{cm}}^{3}$ of air.^(4)^ CTs were obtained before starting treatment and at 15 Gy, 30 Gy, and 45 Gy for two patients and, additionally, 60 Gy for one patient. All CTs have been performed on a helical single‐slice CT scanner. The slice thickness of the CT images was 5 mm for both patients. The in‐plane resolution was 512 × 512 pixels of $0.9766\;\times \;0.9766\;{\text{mm}}^{2}$ for the first patients, and 512 × 512 pixels of $0.8301\;\times \;0.8301\;{\text{mm}}^{2}$ for the second patient.

### B. Prostate contouring — interobserver and intraobserver segmentation variability

The interobserver variability of the manual prostate segmentation was defined by a group of five expert clinicians that, in a blinded fashion, manually contoured repeatedly the prostate in all seven available CTs (0 Gy, 15 Gy, 30 Gy for two patients, and 60 Gy for one patient). The manual segmentation was performed using Eclipse treatment planning system (Varian Medical Systems, Palo Alto, CA), and saved as DICOM‐RT to be used in subsequent analysis. The long‐term intraobserver variability was investigated by asking the same doctors to repeat the prostate segmentation in the available CT data. The average time interval between the delineation of the first and the second set of contours was about six months, in a blinded fashion. For both patients, the segmentation was done for the entire time‐series of CT acquisitions (seven images) resulting in 35 CT sets for the interobserver analysis. For the intraobserver analysis, the segmentation was repeated for the acquisition prior to the RT resulting in a set of 20 CTs.

### C. Distance‐based metrics

Each CT image had a series of five manual segmentations of the prostate, each of them furnished a set of 2D axial contour points defined on subsequent slices of the acquisition. The average center of mass of the prostate (CMP) was estimated from all the contour points of the five segmentations, and each segmentation was reconstructed by using a simple tessellation procedure of the originally defined points. Then, the CMP was used as source point to radially cast 2048 rays, uniformly distributed using a constant angular step in both elevation 0° < 9 < 180° and azimuth 0° < 9 < 360° directions. The average spatial location of the five intersection points between the segmented surfaces and each ray casted from the CMP was then used to estimate an average segmentation surface. At the same time, these intersection points, along each casted ray, define an estimate of the local interobserver variability (within the analyzed CT image). By putting together all these local estimates, we expressed globally the interobserver segmentation variability in terms of standard deviations of contour displacements within the same CT image, as delineated by the group of five clinicians.

In a similar local manner, the long‐term intraobserver variability was expressed in terms of standard deviation of the contour displacements distribution estimated from the long‐term repeated manual segmentations of the five clinicians on the two available CTs prior RT.

### D. Image registration and contour propagation

HERA was used to estimate and to correct for the plasticity of the pelvic structures between subsequent CT scans.^(8)^ HERA optimized an affine transformation (displacement, rotation, skew, and scaling) and an elastic deformation field between the initial CT scan (prior to RT) and each subsequent CT scan (during RT) of each patient. These transformations (affine and elastic) were used eventually to propagate the prostate contour, as defined on the initial CT scan of each patient, prior to RT. The registration procedure used image intensity cross‐correlation as similarity measure, as all images were acquired in the same modality (i.e., CT). In addition, to limit the influence of out of interest pelvic tissues and structures, we restricted the registration process to the prostate surrounding region.

The propagated contours were characterized by using the same ray‐casting strategy as for the evaluation of the intra‐ and interobserver manual segmentation variability.

### E. Evaluation of the contour propagation effectiveness

To evaluate the precision of image registration as a propagation tool, we compared the propagated contours of the prostate with the segmentations obtained manually. We used different measures to locally describe and quantify the precision of the registration for each of the five CT data acquired during the RT.

In a first step, the registration‐based contour propagation was compared to the interobserver variability of the prostate segmentation. Therefore, a two‐tailed Student's *t*‐test was used on the null hypothesis that the mean (average) of the propagated contours is inside the confidence interval of the mean of the manually segmented contours, against the alternative hypothesis that the mean of the propagated contours is outside this confidence interval.

In a second step, the registration‐based contour propagation was compared to the intraobserver variability. Having the CT data segmented twice by each doctor, one can compare the two populations of contour deviations originating from the same person — in one case, the contours have been generated by the same person on the same image, and in the other case, the contours have been generated by the same person on two different images and propagated over. Therefore, we compared the deviations in contouring the same image with deviations in contouring different images that are afterwards registered. If the first population describes only the reproducibility in contouring of the observers, the second population represents a complex variability that accumulates the reproducibility in contouring together with the registration/propagation error.

For all tests performed, the CT done at 0 Gy, prior to RT, was chosen as the floating image. The following CTs were considered as reference. As such, the initial contours are always propagated onto the following acquired images.

In all these statistical tests, each population was consisted of 2048 sets, each consisting of five points. A statistical /‐test was performed locally on each sets of points, along each casted ray (the tests were performed 2048 times). For all tests, a p‐value smaller than 0.05 was accepted as significant.

## III. RESULTS

### A. Contour propagation vs. interobserver variability

First, we evaluated the performance of the propagation of the planning contours against the interobserver variability of the prostate segmentation. Table 1 describes the interobserver variability (the 1st row), together with the registration error of contour propagation while using the affine registration (the 2nd row), and while using the elastic registration (the 3rd row). Table 1 summarizes the average of the descriptive statistics for the differences from the mean prostate contour — the mean, the standard deviation, and the maximal distance. The mean deviation was increased by 1.24 mm with the affine registration and 0.64 mm with the elastic registration, in comparison to the segmented registration. But the maximum registration was reduced by 1.38 mm (respectively 2.76 mm) with the affine (respectively elastic) registration. The mean and maximum deviation was the largest in the Y direction (superior‐inferior direction) for affine and elastic registration due to the CT resolution in the Y direction (5 mm). In the anterior‐posterior direction, the mean deviation was small, below 1 mm for the manual, affine, and elastic registrations. Nevertheless, the maximal deviation could be reduced by 3 mm with the affine registration and 3.7 mm with the elastic registration.

**Figure 1:**
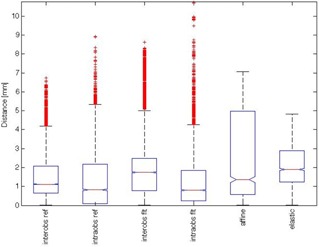
Deviation of inter‐ and intraindividual segmentation and the registration errors. Summary of the intra‐ and interobserver variability of the segmentation and the propagation errors for the target CT performed at 30 Gy. All the deviations are given in absolute values. The box‐plots visualize the local inter‐ and intraobserver variability distribution (along the 2048 rays) together with the contour propagation errors (by affine or elastic transformations) from one set of images that were thereafter registered. The inter‐ and intraobserver segmentation variability is represented on both images: the reference image (a follow‐up image, denoted by *ref)* and the floating image (the planning or initial image, denoted byflt).

**Figure 2:**
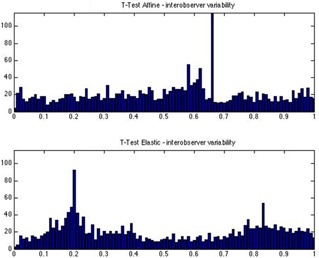
Distribution of the p‐values over the entire surface (2048 points) of the prostate whilst evaluating the performance of contour propagation against the interobserver variability of manual segmentation. The propagation of the planning CT was performed for the CT scan at 30 Gy. The p‐value < 0.05 indicate significant differences and, therefore, propagation failure.

Figure 1 depicts a box‐plot for the local inter‐ and intraobserver variability distribution (along the 2048 rays), together with the contour propagation errors (by affine or elastic transformations) from one set of images that were thereafter registered. The inter‐ and intraobserver segmentation variability is represented on both images — the reference image (a follow‐up image) and the floating image (the planning or initial image). The results showed a slight increase of the median deviation with the affine and elastic registration, in comparison with the manual segmentation. A decrease of the maximum deviation is therefore achieved with the affine and elastic registration. The intraobserver deviation could be reduced by more than 3 mm with the affine registration and by more than 5 mm with the elastic registration. Figure 2 depicts an example of distribution of the p‐values over the entire surface of the prostate. The p‐value represents the goodness of fit of two populations of contours formed on one side by the manual segmentations of the prostate in the currently analyzed image and, on the other side, the affine and elastic segmentations propagated from the planning CT. The values p ≥ 0.05 indicate a failure to propagate the planning contours, which is equivalent with a rejection of the null hypothesis that stated that the two populations are similar. In the present case, 95.5% (respectively 97.4%) of the 2048 sets of points had a p > 0.05 for the affine (elastic) registration.

**Table 1:** Segmentation variability. Mean segmentation variability averaged over all data along the x‐axis (patient's right‐left), z‐axis (patient's anterior‐posterior), and y‐axis (patient's superior‐inferior), and the resulting 3D distance showing the mean, the standard deviation, and the maximum difference for interobserver segmentation, and affine and rigid registration. All measures are given in mm

	*Mean*											
	*X‐mean*	*X‐std*	*X‐max*	*Z‐mean*	*Z‐std*	*Z‐max*	*Y‐mean*	*Y‐std*	*Y‐max*	*Mean*	*Std*	*Max*
Segment	0.56	0.75	6.40	0.57	0.74	6.73	1.04	1.19	7.89	1.52	1.40	9.50
Affine	0.87	0.92	4.05	0.85	0.85	3.74	2.11	2.29	7.60	2.76	2.26	8.12
Elastic	0.64	0.64	3.31	0.64	0.62	2.97	1.69	1.91	6.52	2.16	1.86	6.74

**Figure 3:**
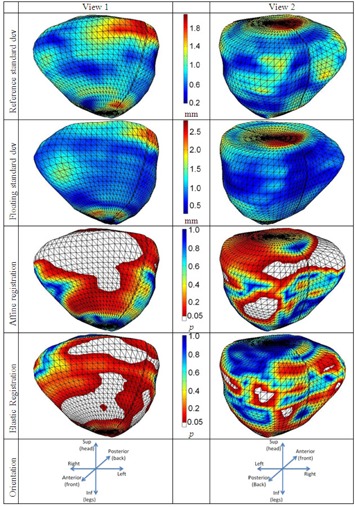
Contour propagation vs. interobserver variability. The surface distribution of the interobserver variability, defined as standard deviation of the segmentations done by five doctors at 30 Gy (the first row) and on the floating data at the time prior to treatment (the second row). The distances are represented in mm. The distribution of the p‐values onto the prostate after affine (the third row) and elastic (the fourth row). The white parts are marking the regions where the contour propagation failed.

Figure 3 illustrates for the same set of images the distribution of various statistics over the segmented surface of the prostate. The interobserver variability in the segmentation of the prostate is the largest in the superior part of the prostate. In this region, the standard deviation was larger than 1.8 mm in the reference images and larger than 2.5 mm on the floating images. The same variability was observed for the left part of the prostate. However, on the right side, the standard deviation was the smallest, under 0.6 mm. The 3rd and 4th rows in Fig. 3 depict the spatial distribution of the p‐values of the contour propagation when using the affine and the elastic registration. The white patches represent those regions where the propagation of the planning contours failed (p < 0.05 ). These regions locate in the anterior direction for the affine and elastic registration and on the right direction for the affine registration. In these regions the interobserver variability is small, less than 1 mm. Small misregistrations had major influence on the statistical tests, were p‐values smaller than 0.05 were observed.

### B. Contour propagation vs. intraobserver variability

**Figure 4:**
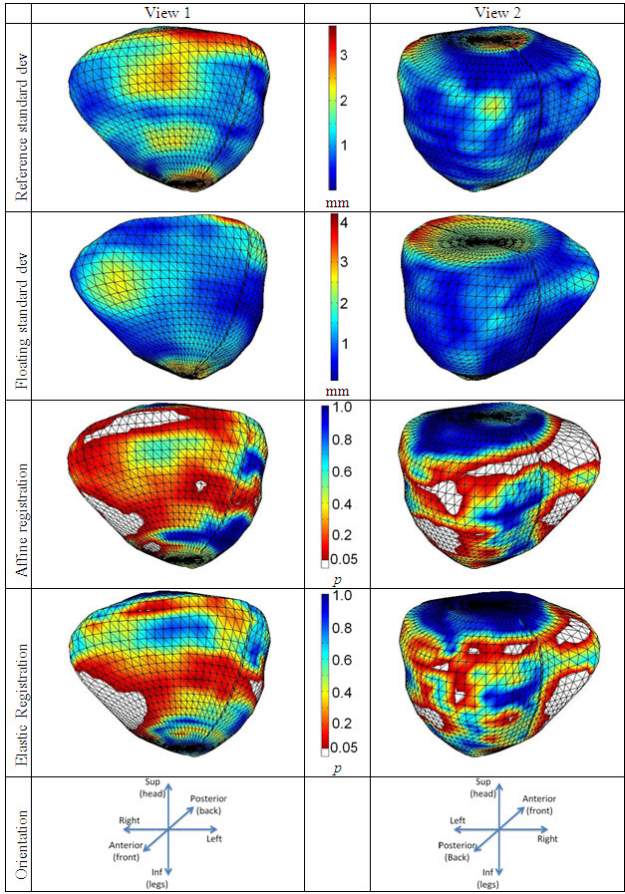
Contour propagation versus intraobserver variability. The first two rows depict the intraobserver variability onto the currently analyzed data (CT performed after 30 Gy) and onto the planning data (CT performed). The following two rows depict the p‐values distributed on the average surface of the prostate. The white parts represent the regions where the standard deviations of the intraobserver variability is smaller than the propagated variability.

A next stage of our study was to examine the performance of the planning contours' propagation against the intraobserver variability. By using the same technique of showing the distribution of various statistics over the segmented surface of the prostate, Fig. 4 depicts the spatial distribution of the intraobserver variability over the reconstructed average surfaces of both planning and target CT on the first two rows. The intraoberver variability is large in the superior and inferior part of the prostate, with a standard deviation up to 4 mm. In the other regions, the standard deviation is below 2.5 mm. The 3rd and 4th rows of Fig. 4 show the spatial distribution of the p‐values on the prostate surface. The registrations are very good in the superior and inferior part of the prostate, while in the right, left, and posterior directions, the propagation failed (the white patches with p < 0.05). Here again, the registration failed in regions were intraobserver variability is smaller than 1 mm.

## IV. DISCUSSION

Intertreatment target volume variability is a well‐recognized source of error in external beam radiotherapy.^(9)^ Repeated 2D or 3D imaging prior to dose delivery has been used to ascertain target coverage and to spare neighboring structures.^(10)^ Currently, clinical standards use rigid positioning verification approaches, basing mostly on 2D and, more recently, on 3D structure matching techniques.^(11)^ Adaptive treatment taking structure deformation into account improves dose distribution of photon‐based treatments.^(12)^

In the present study we show that, by using a registration algorithm as a propagation tool, an automated 3D computer‐based target volume adaptation over a prolonged treatment compares favorably with expert‐derived target reassessment for the purpose of adaptive planning. Target volume propagation using deformable imaging registration for adaptive RT bears several potential advantages, especially the possibility to reduce the internal target margin to close to zero.^(13)^ Imaging systems for target position verification systems, such the Calypso System (Calypso Medical Technologies, Seattle, WA) or the ExacTrac (BrainLAB, Feldkirchen, Germany), allows reducing the irradiated target volume margins to 1 mm if imaging of the prostate localization is used every 15 seconds.^(14)^ In the case of high dose applications, for example prostate, saving millimeters in the posterior margin could be clinically beneficial for the rectum, especially at dose levels exceeding 75 Gy.^(15)^ The reduction of the posterior margin of the planning target volume results in a reduction of the dose applied to the rectum and makes dose escalation feasible. On the anterior direction, a reduction of the margin would also reduce the dose to the pubic symphysis, which is in close proximity to the PTV.

The inter‐ and intraobserver deviations were greater or similar to the variance of the population formed by deviations in contouring for the elastic region in a very large portion of the prostate. Furthermore, elastic registration‐based contour propagation performed better than manual recontouring of the prostate in all three spatial directions with respect to the maximal deviation. The maximal deviation could be reduced using the elastic registration, in comparison to the interobserver variability. The largest deviation was observed in the superior‐inferior direction. This is probably due to the CT resolution in the superior‐inferior direction, which was 5 mm in comparison to 0.8301 mm and 0.9766 mm for the two patients in the left‐right and anterior‐posterior directions. By reducing the slice thickness, a decrease of the mean and maximal deviation could be achieved for the affine and elastic registration.

The affine and rigid registration failed in regions where inter‐ and intraobserver variability was small (< 1 mm) (Table 1). This difference was significant but not clinically relevant, since we are below the setup accuracy.

The elastic registration performed better than the affine adjustment. This improvement will have a direct impact when online reoptimization is used.^(9)^ Indeed, an improvement of the accuracy of the registration will have a direct impact on the margins for the target and, therefore, on the dose to the organ at risks.

## V. CONCLUSIONS

HERA‐based nonrigid prostate volume reassessment using repetitive CT during radiotherapy for the purpose of position verification, target volume adjustment, and online plane reoptimization allows the clinician to minimize PTV margins. Elastic target volume propagation is a feasible and attractive strategy which merits clinical implementation in the treatment workflow and verification of its utility for the purpose of adaptive RT planning.

## ACKNOWLEDGMENTS

We are indepted to colleague physicians Drs. G. Ballerini A. Franzetti, F. Herrera, G. Pesce, and A. Richetti for helping with the target volumes. Supported in part by the Cancer League of Zurich and the Radium Fund, University of Zurich, Switzerland (to I.F.C.).
